# Exposure to selected preservatives in personal care products: case study comparison of exposure models and observational biomonitoring data

**DOI:** 10.1038/s41370-018-0104-3

**Published:** 2018-12-05

**Authors:** Lesa Aylward, Giulia Vilone, Christina Cowan-Ellsberry, Jon A. Arnot, John N. Westgate, Cian O’Mahony, Sean M. Hays

**Affiliations:** 1 Summit Toxicology, LLP, Falls Church, VA USA; 2 Creme Global, Dublin, Ireland; 3 CE2 Consulting, LLC, Cincinnati, OH USA; 4 ARC Arnot Research & Consulting, Toronto, ON Canada; 5 Summit Toxicology, LLP, Bozeman, MT USA

**Keywords:** Personal care products, Preservatives, Exposure models, Biomonitoring, Triclosan, Parabens

## Abstract

Exposure models provide critical information for risk assessment of personal care product ingredients, but there have been limited opportunities to compare exposure model predictions to observational exposure data. Urinary excretion data from a biomonitoring study in eight individuals were used to estimate minimum absorbed doses for triclosan and methyl-, ethyl-, and n-propyl- parabens (TCS, MP, EP, PP). Three screening exposure models (European Commission Scientific Commission on Consumer Safety [SCCS] algorithms, ConsExpo in deterministic mode, and RAIDAR-ICE) and two higher-tier probabilistic models (SHEDS-HT, and Creme Care & Cosmetics) were used to model participant exposures. Average urinary excretion rates of TCS, MP, EP, and PP for participants using products with those ingredients were 16.9, 3.32, 1.9, and 0.91 μg/kg-d, respectively. The SCCS default aggregate and RAIDAR-ICE screening models generally resulted in the highest predictions compared to other models. Approximately 60–90% of the model predictions for most of the models were within a factor of 10 of the observed exposures; ~30–40% of the predictions were within a factor of 3. Estimated exposures from urinary data tended to fall in the upper range of predictions from the probabilistic models. This analysis indicates that currently available exposure models provide estimates that are generally realistic. Uncertainties in preservative product concentrations and dermal absorption parameters as well as degree of metabolism following dermal absorption influence interpretation of the modeled vs. measured exposures. Use of multiple models may help characterize potential exposures more fully than reliance on a single model.

## Introduction

Risk-based assessment of chemicals requires consideration of both intrinsic hazard and exposure potential. Exposures to chemicals may occur via far-field pathways, that is, long-range transport and deposition into environmental media and subsequent human contact. However, for chemicals used in consumer and personal care products, population exposures are generally dominated by near-field exposures, and a variety of exposure models provide algorithms and input assumptions for estimating such exposures [1–3].

The US EPA recommends a tiered approach to assessing exposure to chemicals, progressing from screening-level assessments to more refined and sophisticated assessments as the needs of a given context dictate [4]. There are several approaches to defining tiers with respect to exposure information and models (e.g., [5]). When multiple sources of exposure to a chemical are likely, as in the case of preservatives in personal care products, an aggregate assessment approach is appropriate. The Tiered Aggregate Exposure Assessment Project (TAGS) [6] describes one tiered framework of increasing refinement in aggregate exposure assessment for a population (description below excerpted from [7]):

- Tier 1: Aggregate worst-case exposures for each source of the substance (i.e., use multiple upper-boundary parameter estimates). Such estimates are not intended to represent realistic exposure for the entire population, but rather to provide a conservative bound on potential exposures.
- Refined tier 1: Estimate roughly the realistic exposure in a population. This involves estimating the average exposure as well as lower and upper bounds of exposure in the population.
- Tier 2: Make a detailed estimation of the realistic exposure in the population, including a detailed assessment of the potential distribution of exposures within the population.

In general, a tiered approach progresses from relatively more conservative or upper bound assumptions towards more realistic and representative values grounded in data, where available. Exposure models of varying degrees of refinement are available for assessing potential exposures due to personal care product use. However, there have been limited opportunities to evaluate predictions of these models against observational data for absorbed doses following use of such products. Several studies have conducted case studies in which distributions of predicted exposure levels have been compared to population biomonitoring data for a variety of ingredients of personal care products [7–10]. A recent observational study funded by the European Chemical Industry Council Long-Range Research Initiative (CEFIC LRI) examined variations in urinary concentrations over 6 days of selected ingredients in personal care products that were used by eight volunteers [11]. The concentrations of methyl-, ethyl-, and n-propyl- parabens and triclosan were measured in every urine sample. The data allowed the absorbed dose resulting from the combined use of the personal care products to be estimated. The goal of this analysis is to use this dataset to examine the performance of various screening-level and higher tiered exposure models in predicting actual exposures (absorbed dose) from use of such personal care products.

## Methods

### Biomonitoring data and derived estimates of absorbed dose

#### Dataset description

The urinary biomonitoring data used in this analysis was collected as part of a CEFIC-funded project, “HBM [Human Biomonitoring]-4-VITO: Understanding inter- and intra-individual variability in HBM spot samples.” The dataset and its collection are described in detail in previous publications [11, 12]. Briefly, a convenience sample of eight volunteers in Belgium participated in an observational study over a 6-day period in the autumn of 2012. Their personal care products were inventoried and the ingredient lists scanned for 15 target organic compounds (a suite of parabens, triclosan, triclocarban, bisphenol A, and benzophenones 1, 3, and 8). The participants maintained diaries of times of product use and other factors (meals, smoking, etc.). Product containers were weighed before and after the observation period to ascertain total product use. A two-day “intervention” period was included in the study, during which their personal care products were replaced with products that did not contain any of the target compounds. Over the period of the study, every urine void was collected, time and volume measured, and aliquot preserved for analysis, for a total of 352 samples. The target compounds were measured in each urine sample.

Four preservative ingredients were detected with high frequency in the urine samples: methylparaben, ethylparaben, n-propylparaben, and triclosan (MP, EP, PP, and TCS, respectively), with detection frequencies of 100%, 93.2%, 70%, and 79.5%, respectively [11]. Urinary analysis methods included a deconjugation enzyme treatment resulting in measurement of parent compounds whether present as free compound or in conjugated form, but did not quantify hydrolysis products or oxidative metabolites [11]. Inspection of the urinary concentration vs. time profiles indicated that peaks could clearly be associated with product uses, and concentrations declined significantly to low or non-detectable levels during the two-day intervention period. As a result, the data are consistent with the assumption that personal care product use was the main source of exposure to the four compounds during the 6-day period, representing 4 days of product usage, and that these compounds are rapidly eliminated from the body [11].

#### Estimation of minimum absorbed doses

For each participant, the average daily minimum absorbed dose (D_min,_ µg/kg/day) of parent compound was calculated based on the amount of parent preservative excreted in urine over the 6 days of observation, divided by the participant bodyweight (BW) and 4 days of product use:

1 $$D_{\mathrm{min}} = \frac{{\mathop {\sum }\nolimits_{i = 1}^n C_iV_i}}{{BW \ast 4}}$$

where *n* is the number of urine voids collected for the individual and *C*_*i*_ and *V*_*i*_ are the analyte concentration and void volume for each void, respectively. Non-detected concentrations were imputed with zero, consistent with the goal of estimating minimum absorbed dose.

While exposures occurred for only 4 of the 6 days, the analyte measurements were summed for all 6 days, allowing capture of the excretion during non-exposed days of compounds applied on exposed days. The half-lives of urinary elimination for the parabens are generally less than 8 h and ~11 h for triclosan [13, 14]. Inclusion of the mass of eliminated parent compounds on the two intermediate days of non-use of products captures some carryover elimination of the compounds from use on the previous day. Carryover from days of use prior to the first observational day is also included in the sampling data, even though product use is not inventoried for that day. This is balanced by no collection of urinary elimination following the observational period. Thus, the total mass excreted over the six observational days generally represents exposures from 4 days of product use, so the excreted mass is normalized to 4 days rather than 6.

The average amount of parent compound excreted daily represents the minimum absorbed dose because the urinary analysis does not quantify metabolites produced via hydrolysis or oxidative metabolism following dermal or oral absorption of parent compounds, nor does it account for any compound retained in the body or potential fecal excretion. The calculation of average daily minimum absorbed doses potentially yields 32 values, four preservatives for each of the eight participants. An upper bound on potential exposures can also be estimated using urinary excretion fractions observed following oral exposure [13, 14]. Measured and estimated urinary excretion fractions for these compounds are 17.4%, 13.7%, 9.7%, and 54% for MP, EP, PP, and TCS, respectively. These urinary excretion fractions are based on observations following controlled oral dosing, which entails substantial first-pass metabolism. However, in this observational study most exposure was to compounds applied dermally, which would not entail first-pass gut metabolism. As a result, calculated maximum absorbed doses using these urinary excretion fractions are likely to substantially overestimate total systemically absorbed parent compound for this study, but are presented as a useful upper bound on the potential systemically absorbed doses associated with the observed urinary excretion.

### Models and assumptions

A number of models were considered for inclusion in the evaluation of modeling outputs presented here based on availability and knowledge of the participating authors (Table 1). These models represent a range of refinement, assumptions and sophistication. In the context of the TAGS project tiered approach framework, these models range from Tier 1 to refined Tier 1 to Tier 2. Briefly, the basic underlying calculation of systemic exposure dose (SED) [15] for a chemical in a product is:

**Table 1:** Models considered for inclusion in the evaluation

Model	Description	Comments
Tier 1 Models		
ConsExpo	Publicly available exposure model from the Dutch National Institute for Public Health and the Environment (RIVM; http://www.rivm.nl/en/Topics/C/ConsExpo). Includes both a deterministic and probabilistic capability	Deterministic mode was included. Probabilistic mode was not included due to the lack of default distributions for key simulation parameters
EC SCCS Notes of guidance algorithms	EC SCCS (2016) document presents comprehensive guidance for assessment of ingredients for personal care products. Algorithms are presented for estimating exposure from use of a range of products. Also includes an aggregate scenario for assessment of preservative ingredients	Included two calculations: (a) product-specific algorithms for each individual for their uses of products with the preservative ingredients, and (b) calculation using the aggregate preservative algorithm
RAIDAR-ICE	RAIDAR-ICE is an extension of the Indoor Chemical Exposure Classification/Ranking Model (ICECRM) providing a framework for high-throughput and screening-level aggregate exposure and risk assessment. For personal care products applied directly to the skin (hands or “rest of body”) the user can define if the chemical is a leave-on product (e.g., hand cream or deodorant) or a wash-off product (e.g., shampoo, soap)	Included. The model was implemented in two forms. The “Tier 1” implementation uses the predictions of dermal absorption based on physical-chemical properties and QSARs. The “Empirical” implementation uses the empirical assumptions regarding dermal absorption fractions. Product application rates were usually taken from the EC SCCS default exposure scenarios for each product category
EPA Consumer Exposure Model (CEM)	Model designed to assist in TSCA evaluations for consumer products (https://www.epa.gov/tsca-screening-tools/consumer-exposure-model-cem-version-20-users-guide)	Not included; no modules designed to evaluation personal care product uses
ECETOC Targeted Risk Assessment (TRA) tool	Exposure assessment component of TRA designed for application under REACH. However, no specific scenarios for personal care product exposure assessment are included	Not included; no modules designed to evaluation personal care product uses
Refined Tier 1 or Tier 2 models		
SHEDS-HT	Publicly available high-throughput version of EPA Stochastic Human Exposure and Dose Simulation model (https://www.epa.gov/chemical-research/stochastic-human-exposure-and-dose-simulation-sheds-estimate-human-exposure)	Included
Creme Care & Cosmetics	Probabilistic model built upon a habits and practices database of over 36,000 consumers from Europe and the United States. Designed to be a higher-tier exposure assessment tool. Proprietary model	Included
Probabilistic Aggregate Consumer Exposure Model (PACEM)	Population simulation tool designed to integrate with ConsExpo to produce population distribution estimates of exposure	Not included. Model is not publicly available and RIVM researchers were unavailable to provide model runs for inclusion in this analysis

SED (μg/kg/d) = *A* (μg/kg/d) *x R* × *C* × *D* [2]

where *A* is the daily application rate of product normalized to body weight and includes the *amount* and *frequency* of product used (often referred to as habits and practices information); *R* is the retention factor, representing the amount of product remaining on skin after application; *C* is the concentration fraction of the chemical of interest in the product (often proprietary); and *D* is the absorption efficiency of the chemical (expressed as a fraction). Alternatively, some models estimate dermal uptake via consideration of physical/chemical properties relevant to dermal uptake and time of retention of the product on a defined surface area of skin.

Five models were implemented to estimate exposure for each of the eight participants in the observational study. Three of the models are Tier 1 models, with deterministic inputs and outputs: ConsExpo from the Dutch Ministry of Health and Environment (RIVM, http://www.rivm.nl/en/Topics/C/ConsExpo), the European Commission Scientific Committee on Consumer Safety (SCCS) notes of guidance algorithms, in both product-specific and aggregate preservative modes [15], and the Risk Assessment IDentification And Ranking-Indoor and Consumer Exposure (RAIDAR-ICE v.0.803; available at www.arnotresearch.com/models/) model. ConsExpo can also be implemented in a probabilistic mode to obtain higher-tier estimates of exposure. This implementation was not used in this exercise because default input distributions are not provided and must be generated by the user, an exercise outside the scope of this analysis. RAIDAR-ICE is an extension of the Indoor Chemical Exposure Classification/Ranking Model (ICECRM) [16]. It includes direct exposure pathways (inhalation, dermal, ingestion) relevant for many personal care products, with inclusion of the SKINPERM QSAR model for predicting skin permeability coefficient [17]. Finally, two probabilistic models that employ refinements in exposure assumptions through the use of distributional inputs and which provide predictions of population distributions of exposure as outputs were also evaluated: US EPA’s Stochastic Human Exposure and Dose Simulation – High Throughput (SHEDS-HT; available at https://www.epa.gov/chemical-research/stochastic-human-exposure-and-dose-simulation-sheds-estimate-human-exposure) [18], and Creme Care & Cosmetics, a proprietary software package based on a published model (Creme C&C) [19].

Each model was run using its defaults for product application amounts and frequencies and retention times or retention factors for the product use profiles for each participant in the observational study. For example, if a participant reported using shampoo containing methylparaben and propylparaben as well as shower gel and toothpaste with triclosan, those four product/ingredient combinations were modeled for that individual, applying the model default values for product application rates and retention factors for those products. The lone exception to this was for the SCCS default aggregate preservative scenario, which assumes a set exposure scenario of 17 personal care products. including rinse-off skin and hair cleansing products, leave-on skin and hair care products, make-up products, and oral care cosmetics.

Estimates of chronic daily average absorbed doses in μg/kg-d for each combination of participant (with their reported products used) and ingredient were generated from each of the models. In this evaluation it was assumed that direct exposures from the documented sources are the only relevant exposure pathways, that is, that no other sources of the preservatives contributed to the observed urinary excretion of these compounds, consistent with the previous evaluation of the urinary biomonitoring data [11].

The probabilistic models (SHEDS-HT and Creme C&C) were run for the exposed population only, so that the distributions of estimated absorbed doses did not include non-exposed individuals. The Creme C&C model was run for participant-specific aggregate scenarios. For example, Participant 1 used shampoo, shower-gel, day-cream, and body-lotion containing MP, so the model was run for a population using only these four products containing MP. For SHEDS-HT, this required that product use frequency be set at a point estimate rather than as a distribution, which is the normal default in the model, because SHEDS-HT is a one-day model. Thus, when a distribution is applied for frequency of exposure, some iterations of the model result in zero exposure; exposures are not averaged over multiple days of use.

All of the models required an input for the ingredient concentration (*C* in Eq. 2). The regulatory maximum limits for each ingredient in force in Europe at the time of the observational study were 0.4% for methyl- and ethyl-paraben (EC SCCS 2014); 0.19% for n-propylparaben (EC SCCS 2014); and 0.3% for triclosan (EC SCCP 2009). Recent reviews of available product data suggest that a value of 0.1% is a more typical concentration for these preservatives, but that concentrations vary by product type and specific product [20]. Because absorbed dose is a linear function of ingredient concentration for all models, the modeling results can be adjusted to evaluate alternative concentrations. For this effort, a scenario assuming a constant 0.1% concentration for all preservatives in all products was assessed.

The EU SCCS and Creme C&C models required an input for dermal absorption fraction (*D* in Eq. 2). For the parabens, 3.7% dermal absorption was used, a value identified following review of the literature and selected in the EC SCCS evaluation of parabens [21]. For triclosan, the dermal absorption fraction was estimated at 7.7% for deodorant, 7.2% for shower gel, and 11.3% for toothpaste, again, based on the review of the literature and current EC safety assessment [22]. The remaining models do not require inputs for dermal absorption fraction because dermal uptake is estimated using physical/chemical properties. This feature is an advantage for screening thousands of “data-poor” chemicals, many of which do not have empirical estimates of *D*. RAIDAR-ICE also allows the user to provide an empirical value for *D*, if available, to override the default QSAR calculation. In this case study RAIDAR-ICE was run with and without empirical estimates of *D* providing an indication of the sensitivity of exposure calculations with different tiers of chemical-specific absorption efficiency information.

Oral absorption following incidental ingestion for the toothpaste scenario was assumed to be 100%. Retained ingested amounts were estimated as 5% of the product used, except in the ConsExpo model, in which this parameter is set at 0.08 g.

### Statistical evaluation of model predictions compared to biomarker-derived dose estimates

A number of approaches were used to compare the predictions from each model to the calculated minimum absorbed doses based on the biomonitoring data and to assess the overall performance of each model. Assessing the performance of models typically involves examining the correlation and correspondence of predicted values compared to observational data, as well as an examination of the degree and direction of predictions (over-predictions and under-predictions) relative to the measured values. In the case of these models, the particular patterns of over-prediction and under-prediction for each model are of specific interest. These models may be applied in a variety of contexts, including regulatory or safety assessments or individual or population-wide exposure assessments. In some contexts, such as safety assessments, consistent over-estimations of exposure may be acceptable or preferable. In other contexts, such as higher-tier exposure assessments, a more balanced performance of the model with better accuracy may be important.

For this assessment a combination of approaches was used to provide an integrated assessment of the model predictions across all four preservatives. Spearman rank correlation coefficients were calculated for each set of model predictions. In addition, an analysis of the fraction of predictions from each model that fall within a factor of three and ten of the measured value was conducted, detailing both under-predictions and over-predictions, providing a perspective on the proportion of predictions that fall within ranges that are relevant in the context of risk assessment.

## Results

### Product use and calculated minimum and maximum absorbed doses

The eight participants used various combinations of seven product types containing one or more of the four preservatives: toothpaste, shampoo, shower gel, deodorant, shaving cream, day cream, and body lotion (see Table 2). There are 32 possible aggregate exposure combinations (four ingredients for each of eight participants). However, based on the pattern of product/ingredient use by each participant, there are actually only 25 participant/ingredient combinations that are relevant for the dataset because some participants did not use any products containing one or more of the target ingredients. Specifically, participants 3, 4, 6, 7, and 8 did not use any products containing ethylparaben, and participants 3 and 4 did not use any products containing triclosan (Table 2).

**Table 2:** Product and ingredient use patterns by participant

Product	Toothpaste	Shampoo	Shower gel	Deodorant	Shaving cream	Day cream	Body lotion
Participant 1							
MP		X	X			X	X
EP		X				X	
n-PP			X				X
Triclosan	X						
Participant 2							
MP		X	X		X	X	
EP						X	
n-PP			X		X		
Triclosan	X						
Participant 3							
MP	X						
EP							
n-PP	X						
Triclosan							
Participant 4							
MP	X	X					
EP							
n-PP	X						
Triclosan							
Participant 5							
MP						X	
EP						X	
n-PP						X	
Triclosan			X	X			
Participant 6							
MP		X	X		X		
EP							
n-PP			X		X		
Triclosan	X						
Participant 7							
MP		X	X			X	
EP							
n-PP			X				
Triclosan	X						
Participant 8							
MP		X	X			X	
EP							
n-PP			X				
Triclosan	X						

The average daily excretion rates of parent preservative compounds by each of the participants in the observational dataset are presented in Table 3. These excretion amounts include parent compound present in urine either as free compound or as conjugated compounds (glucuronides or sulfates). These amounts represent the average daily minimum absorbed doses of these parent compounds over the four days of product exposure. Additional parent compounds may have been absorbed and subsequently metabolized to metabolites not included in the urinary analysis such as hydrolysis products for the parabens (PHBA and PHHA). In addition, some absorbed compounds could have been excreted via feces. Thus, the quantified excreted parent compound (and conjugates) in urine represents a minimum absorbed dose.

**Table 3:** Average daily excretion of parent preservative compounds over the observational study period. Non-detected concentrations were imputed with zero. Data from [11]

Participant	BW (kg)	Avg. daily excreted, μg/kg-d			
		Methylparaben	Ethylparaben	n-Propylparaben	Triclosan
1	58	12.13	2.29	5.02	35.09
2	92	2.99	2.42	0.04	21.98
3	57	0.63	0.04^a^	0.18	0.01^a^
4	85	1.99	0.18^a^	0.52	0.00^a^
5	66	4.26	0.99	1.18	17.23
6	95	0.18	0.05^a^	0.01	6.42
7	63	0.80	0.09^a^	0.30	10.66
8	78	3.57	0.50^a^	0.02	10.14
Mean (SD) users:	3.32 (3.85)	1.90 (0.79)	0.91 (1.70)	16.92 (10.5)	
Mean (SD) non-users:	NA	0.17 (0.19)	NA	0.01 (0.01)	
Mean (SD) all:	3.32 (3.85)	0.82 (1.00)	0.91 (1.70)	12.69 (11.83)	

*NA* not applicable

^a^No reported use of personal care products with this preservative

The average daily excretion rates were highest for triclosan, followed by methylparaben, propylparaben, and ethylparaben, with means of 12.69, 3.32, 0.91, and 0.82 μg/kg-d, respectively. Five individuals did not use products containing ethylparaben; when they are omitted from the calculation, average daily excretion of ethylparaben rises to 1.9 μg/kg-d. Two individuals did not use products containing triclosan. When they are omitted, average daily urinary excretion of triclosan for those persons using triclosan-containing products is 16.9 μg/kg-d (Table 3). The urinary data show that excretion is very low for persons not using personal care products containing the target ingredients, indicating that for the participants during the time period of this study, personal care product use was the dominant pathway for exposure to these four preservatives.

Maximum systemically absorbed doses, calculated by application of the oral urinary excretion fractions were also calculated and are presented in Table 4. As discussed in the Methods section, these likely overestimate dermally absorbed parent compounds due to the lack of first-pass hepatic metabolism by this route in comparison with the oral exposure conditions used to measure the urinary excretion fractions. However, these values may provide an estimate of plausible upper bounds of potential systemically absorbed compounds under conditions of dermal application of the personal care products.

**Table 4:** Observed minimum and maximum absorbed doses (μg/kg-d) and corresponding predicted absorbed doses by participant and ingredient from each model, assuming preservatives are at their maximum allowed concentration (0.4% for MP and EP, 0.19% for PP, and 0.3% for triclosan). Spearman rank correlation coefficients between model predictions and minimum and maximum absorbed dose estimates are also presented

Person/chem	Min_abs_dose	Max. abs. dose^a^	ConsExpo	SCCS default	SCCS product-specific	RAIDAR-ICE Tier 1	RAIDAR-ICE empirical	SHEDS P05	SHEDS mean	SHEDS P95	Creme CC P05	Creme CC mean	Creme CC P95
P1/MP	12.13	69.69	21.46	12.04	5.61	105.00	17.37	0.03	0.85	3.12	0.01	0.78	3.85
P2/MP	2.99	17.16	13.96	12.04	1.94	9.48	2.92	0.01	0.35	1.36	0.01	0.22	0.99
P3/MP	0.63	3.62	2.30	12.04	2.24	1.64	6.55	0.38	2.57	7.42	0.90	3.96	9.56
P4/MP	1.99	11.41	9.97	12.04	2.30	1.84	6.61	0.39	2.55	7.61	0.16	0.99	2.37
P5/MP	4.26	24.50	0.86	12.04	0.89	8.45	2.72	0.01	0.30	1.20	0.22	2.69	9.17
P6/MP	0.18	1.06	13.10	12.04	1.05	1.02	0.19	0.01	0.04	0.16	0.01	0.23	1.04
P7/MP	0.80	4.61	12.86	12.04	1.05	9.45	2.91	0.01	0.35	1.36	0.01	0.23	1.04
P8/MP	3.57	20.52	12.86	12.04	1.05	9.45	2.91	0.01	0.35	1.36	0.01	0.23	1.04
P1/EP	2.29	16.68	8.53	12.04	0.95	6.05	2.69	0.01	0.32	1.27	0.01	0.22	1.05
P2/EP	2.42	17.69	0.86	12.04	0.89	5.93	2.63	0.01	0.31	1.20	0.22	2.69	9.17
P5/EP	0.99	7.23	0.86	12.04	0.89	5.93	2.63	0.01	0.31	1.20	0.22	2.69	9.17
P1/PP	5.02	51.70	12.93	12.04	4.66	89.89	6.91	0.03	0.57	2.20	0.01	1.06	4.53
P2/PP	0.04	0.38	5.43	12.04	0.99	0.82	0.09	0.00	0.04	0.15	0.01	0.38	1.59
P3/PP	0.18	1.89	2.30	12.04	2.24	1.64	3.11	0.38	2.56	7.37	0.43	1.88	4.54
P4/PP	0.52	5.34	2.30	12.04	2.24	1.64	3.11	0.38	2.56	7.37	0.43	1.88	4.54
P5/PP	1.18	12.13	0.86	12.04	0.89	3.09	1.13	0.01	0.31	1.20	0.10	1.28	4.36
P6/PP	0.01	0.12	5.43	12.04	0.99	0.82	0.09	0.01	0.04	0.15	0.01	0.38	1.59
P7/PP	0.30	3.08	4.33	12.04	0.10	0.74	0.06	0.01	0.04	0.15	0.01	0.09	0.21
P8/PP	0.02	0.19	4.33	12.04	0.10	0.74	0.06	0.01	0.04	0.15	0.01	0.09	0.21
P1/TC	35.09	64.98	2.30	22.72	2.40	1.64	4.91	0.38	2.57	7.41	0.67	2.97	7.17
P2/TC	21.98	40.70	2.30	22.72	2.40	1.64	4.91	0.38	2.57	7.41	0.67	2.97	7.17
P5/TC	17.23	31.91	14.80	22.72	1.82	2.54	3.06	0.04	0.39	1.24	0.02	0.22	0.94
P6/TC	6.42	11.90	2.30	22.72	2.40	1.64	4.91	0.38	2.57	7.41	0.67	2.97	7.17
P7/TC	10.66	19.74	2.30	22.72	2.40	1.64	4.91	0.38	2.57	7.41	0.67	2.97	7.17
P8/TC	10.14	18.78	2.30	22.72	2.40	1.64	4.91	0.38	2.57	7.41	0.67	2.97	7.17
Spearman rank correlation coefficients													
Compared to minimum abs. dose:			0.018	0.701**	0.602**	0.378	0.649**	0.438*	0.648**	0.554**	0.355	0.41*	0.320
Compared to maximum abs. dose:			0.054	0.467*	0.521**	0.555**	0.609**	0.353	0.512**	0.441*	0.224	0.312	0.290

*p < 0.05; **p < 0.01

^a^Calculated as the minimum absorbed dose divided by the chemical-specific urinary excretion fraction (see text)

### Model results and evaluation

**Fig. 1:**
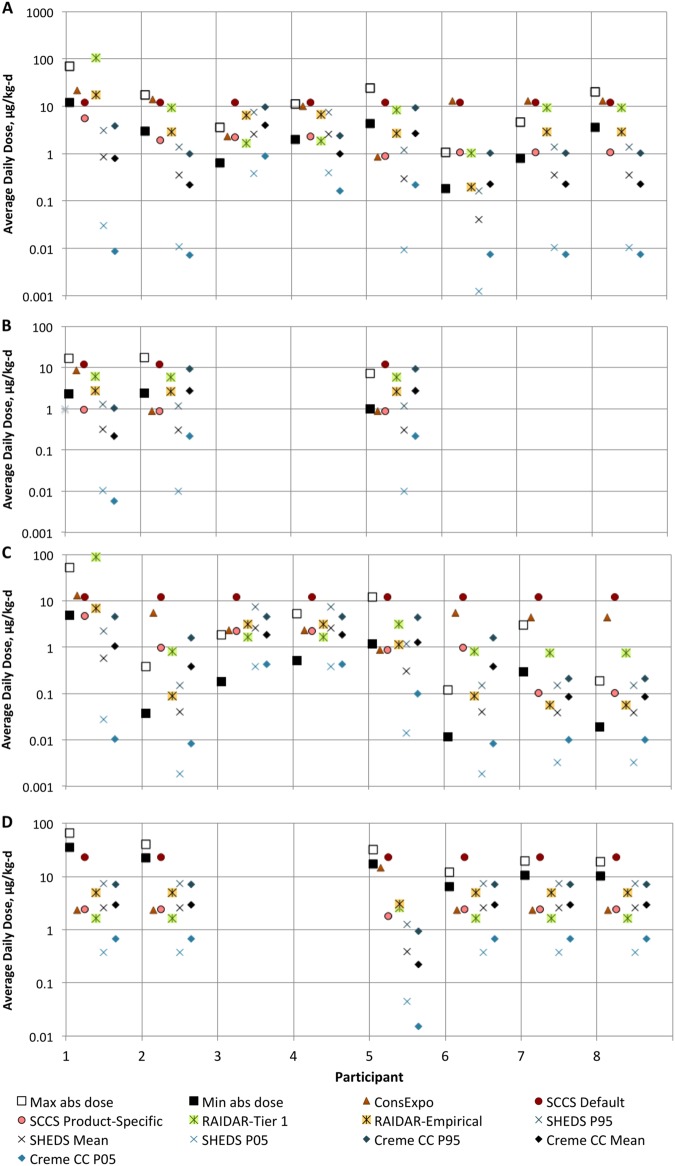
Minimum and maximum absorbed doses calculated from urinary excretion data and modeled doses by participant for **a** methylparaben; **b** ethylparaben; **c** n-propylparaben; and **d** triclosan

The predicted absorbed doses for each participant and ingredient by each model are illustrated in comparison to the minimum absorbed dose and corresponding estimated maximum absorbed dose (which likely overestimates actual absorbed doses) for the 25 relevant participant/ingredient combinations in Fig. 1, assuming 0.1% concentration of the preservatives present in all products. For the two models that provide probabilistic predictions, predictions representing the mean as well as the 5th and 95th percentiles for each participant/ingredient combination are presented. The predicted absorbed doses from each model assuming product preservative concentrations for each participant/ingredient combination are presented in Table 4, along with the estimated minimum and maximum absorbed doses derived from the urinary biomonitoring data.

In general, the highest predicted absorbed doses across all preservative ingredients come from the EC SCCS default aggregate preservative scenario calculations and the RAIDAR-ICE Tier 1 model. The EC SCCS default aggregate preservative scenario assumes that an individual is exposed to the preservative in all of their personal care products [15]. Using this scenario, the predicted absorbed dose is the same across all individuals for a given ingredient and assumed absorption fraction. This scenario is used for the overall risk assessment of preservative compounds used in personal care products in the EU. It is intended to provide a highly conservative evaluation of potential preservative exposure, envisioning potential exposure from a full range of personal care products including rinse-off skin and hair cleansing products, leave-on skin and hair care products, make-up products, and oral care products. For the participants in this observational study, this approach is confirmed to result in estimates of exposure that are generally greater than the observed exposures, which is consistent with the lower number of products used by the participants compared to the default aggregate assumption. The high-throughput screening-level RAIDAR-ICE model using QSAR-derived estimates for skin permeability also produces relatively high exposure estimates compared to the minimum absorbed doses, while the RAIDAR-ICE model parameterized with empirical chemical-specific estimates of dermal absorption efficiency is generally less conservative.

Correlation of model predictions to estimated minimum and maximum absorbed doses was assessed using Spearman rank correlation coefficients (Table 4). The statistical performance of the models varied, and was generally modest. The EU SCCS models, the RAIDAR-ICE model with empirical dermal absorption fraction, and SHEDS-HT mean predictions all were significantly correlated with the minimum absorbed dose estimates with coefficients >0.6. Other comparisons showed generally lower correlations.

**Fig. 2:**
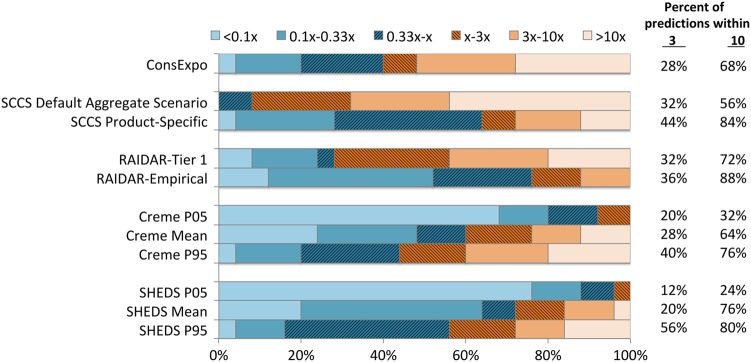
Overall performance of models assuming 0.1% preservative content in all products compared to minimum absorbed doses based on fractions of predictions within a factor of 3, 10, or more than a factor of 10. Under-prediction and over-prediction frequencies are illustrated in blue and orange, respectively. The fraction of predictions within a factor of 3 of the minimum absorbed dose are illustrated with the darkest shading; predictions between factors of 3 and 10 with intermediate shading, and predictions more than 10-fold above or below the observed value are the lightest shading. The text columns to the right present the sum of the percent of predictions within 3- and 10-fold of the minimum absorbed dose

Another way to evaluate the accuracy of the model predictions is to examine the fraction of predictions within a given factor of the absorbed doses estimated based on the urinary biomonitoring data. Figure 2 shows the fraction of total predictions by each model (assuming 0.1% concentrations) within a factor of 3 and within a factor of 10 (that is, a factor of 3 or 10 above or below the minimum observed absorbed doses), as well as the fractions of predictions that are more than 10-fold different from the observed minimum absorbed doses. These are arbitrary values potentially of interest in a risk assessment context. Several of the models resulted in ~30–40% of the predictions within a factor of 3 of the observed minimum absorbed doses. In general, ~55–90% of the predictions were within a factor of 10 (excepting the 5th percentile estimates from the probabilistic models). The patterns of over-prediction and under-prediction are consistent with what would be expected based on the model goals and approaches. That is, the SCCS default aggregate model nearly always over-predicts, and over-prediction is more frequent than under-prediction for the ConsExpo and RAIDAR Tier 1 models as well. For the probabilistic models, predictions at the mean or 95th percentiles tended to provide a greater fraction of predictions within factors of 3 or 10 of the observed data, while lower percentile predictions were less accurate and frequently under-predicted compared to the absorbed dose estimates for these eight participants.

Evaluation of the individual participant/ingredient predictions from the probabilistic models (SHEDS-HT and Creme C&C) via comparison of the observed doses to a single point estimate drawn from the distributions that arise from the modeling (e.g., the 5th or 95th percentile or the mean) is clearly a misapplication of the strength of the probabilistic analyses. Individuals in the study over the short-term and long-term would be expected to have varying habits and practices, even when the same products are being used, and the probabilistic models are intended to recapitulate that intra-individual variation as well as inter-individual variation. Thus, a more pertinent question is whether or not the distributions of model predictions for each of the 25 participant/ingredient combinations encompassed the observed minimum absorbed dose for those combinations. In this respect, the distribution of predictions usually captured the estimated minimum absorbed doses for parabens; however, observed absorbed doses for triclosan frequently were greater than the upper end of the distribution of predictions from both models (Table 5).

**Table 5:** Percentiles of the probabilistic predictions for each participant/ingredient combination at 0.1% preservative concentration corresponding to the observed minimum absorbed dose

Participant/ingredient	Min. absorbed_dose, μg/kg-d	Percentile corresponding to observed minimum absorbed dose	
		Creme	SHEDS-HT
P1/MP	12.13	90–95	>99
P2/MP	2.99	90–95	99
P3/MP	0.63	<5	<5
P4/MP	1.99	25	60
P5/MP	4.26	85	>99
P6/MP	0.18	40	95
P7/MP	0.80	60	90
P8/MP	3.57	90–95	>99
P1/EP	2.29	90	98
P2/EP	2.42	90	99
P5/EP	0.99	35	90–95
P1/PP	5.02	80	99
P2/PP	0.04	20	75
P3/PP	0.18	<5	<5
P4/PP	0.52	<5	10
P5/PP	1.18	65	95
P6/PP	0.01	5	40
P7/PP	0.30	99	>99
P8/PP	0.02	10	55
P1/TCS	35.09	>99	>99
P2/TCS	21.98	>99	>99
P5/TCS	17.23	>99	>99
P6/TCS	6.42	90–95	90–95
P7/TCS	10.66	99	98
P8/TCS	10.14	99	98

*MP* methylparaben, *EP* ethylparaben, *PP* n-propylparaben, *TCS* triclosan

This same pattern was observed generally for the predictions from all of the models (Fig. 1). Five of the six participants exposed to triclosan were exposed only through toothpaste use (participants 1, 2, 6, 7, and 8). The relatively consistent underestimation for triclosan suggests that retention fractions or application quantities of triclosan from toothpaste are generally greater than the assumed values in these models. Three other participant/ingredient combinations were also related only to toothpaste use: participant 3, for methylparaben and propylparaben, and participant 4 for propylparaben. In these cases, the minimum absorbed doses fell at very low percentiles in the model predictions. However, only small fractions of orally ingested parabens are excreted as parent compounds in urine [13]. When the maximum absorbed doses calculated from the urinary excretion fractions for these compounds are considered, the parabens predictions fall much closer to the estimated maximum absorbed doses for the toothpaste users (Fig. 1). This suggests that the underestimation of triclosan exposure via toothpaste may be due to underestimation of retained/swallowed product (retention factor) for some of the participants, but might also be related to greater mass of toothpaste used by some of the participants or to possible underestimation of buccal absorption of triclosan during toothpaste use.

## Discussion

The observational dataset provided 25 calculated minimum absorbed doses corresponding to aggregate exposures for participant/ingredient combinations, and these estimated absorbed doses ranged over more than 3 orders of magnitude. Evaluation of the overall performance of each of the models is challenging due to a number of factors. Both the degree of agreement and the direction of any bias in predictions are of interest in evaluating the model outputs. Making such comparisons is relatively straightforward for the deterministic models; however, for models producing probabilistic output, the evaluation is more complex. In addition, criteria for evaluating model performance depend somewhat on the goal of the modeling. If conducted in a screening, regulatory framework, the goal may be to provide plausible estimates that are unlikely to underestimate actual exposures (e.g., a Tier 1 assessment; [7]). If conducted in the context of a more refined population exposure assessment basis, the goal may be to more accurately predict the distribution of actual exposures in the population (a higher tier assessment).

The models evaluated here generally used similar algorithms to assess exposures (e.g., Eq. 2). Thus, differences in results are mainly attributable to differences in default values for key habits and practices assumptions such as frequency of use, amount of product used, retention factor or retention time, etc. as well as to differences in the dermal absorption prediction algorithms (for those models using this approach). We did not attempt to compare all of these values or distributions used in the various models in this work. However, we did compare the assumptions regarding mean amount of product applied per day from the various models to the observational data on product usage (Table 6). Usage of shampoo and shower gel in the observational data was lower than assumed in any of the models. For other products, the amounts assumed in the modeling generally were in the same range as the average usage rates recorded in the observational data; however, some individual usage rates over the four days of product use were higher than the estimates assumed to be conservative in the Tier 1 model parameterization and subsequent calculations.

**Table 6:** Average product usage amounts, g/d, from models, literature, and observational data [11]

Model	Body lotion	Face cream	Deodorant	Shampoo	Shaving cream	Shower gel	Toothpaste
ConsExpo	8	0.8	5.2	20	2	8.7	–^a^
SCCS [15]	7.82	1.54	1.43	10.46	1.54^b^	18.67	2.75
SHEDS (mean)	8	5	2	12	2	12	6
Creme (mean)	3.63	1.33	2.36	7.59	1.33	8.86	1.55
RAIDAR-ICE ^c^	7.82	1.54	1.43	10.46	1.54	18.67	2.75
Literature values							
Biesterbos et al. [23], mean	3.6	0.4	0.4	2.4	1.3	4.5	2.2
Hall et al. (2007, 2011), mean^d^	4.5	NR	NR	6	NR	11.3	2.1
Observational data, this study							
Measured product use, mean (SD), g/d	8.65 (NA)	1.37 (0.94)	6.97 (NA)	5.75 (2.4)	1.71 (0.08)	6.87 (4.3)	2.35 (1.96)
Number of participants using:	1	5	1	5	3	6	6

*NR* Not reported, *NA* Not applicable—only one participant used this product so no standard deviation was calculated

^a^Model uses an input for the amount orally retained and ingested. Amount of product assumed used is not given directly

^b^Not specified in SCCS documentation [15]. Assumed same parameters as face cream

^c^Input values were adopted from [15]

^d^From references [24, 25], as reported in [23]

Two major uncertainties affect the model estimations of absorbed dose. First, actual concentrations of the preservatives in each of the products used are not known. Changes to this parameter (i.e., “*C*” in Eq. 2) are linear in the model calculations. The regulatory maximum concentrations in force in Europe at the time of the observational study was 0.4% for methyl- and ethyl-parabens; 0.19% for n-propylparaben; and 0.3% for triclosan. A 2008 review by the Cosmetics Ingredient Review [20] collected available information on preservative concentrations in various classes of personal care products [20]. At that time, a concentration of about 0.1% appeared to be a more reasonable estimation of likely average values, and that value was used in this assessment. However, the actual concentrations of the preservatives likely varied by specific product and could have been higher or lower.

Second, the dermal absorption fraction was not known for any of the preservative/product combinations, and dermal absorption is known to vary substantially by vehicle and application conditions such as retention time [20]. Three of the models (EC SCCS, RAIDAR-ICE in the empirical mode, and Creme C&C) require input of an absorption fraction for running the model; while by default SHEDS-HT and RAIDAR-ICE predict absorption rates using QSARs based on physical-chemical properties. Values selected in previous EC SCCS evaluations of these preservatives were input in all three models (see above). Because of their selection in a regulatory safety assessment framework, these values might be expected to be conservative (i.e., tend to overestimate systemically available dose following dermal absorption). However, these values are unlikely to be accurate for all dermally applied products, since the available literature clearly shows substantial differences in absorption depending on the vehicle [20]. Comparison of the two sets of RAIDAR-ICE output highlights the sensitivity of all exposure model calculations to the absorption parameter (“*D*”) in Eq. 2. Differences in QSAR predictions and empirical estimates for *D* for parabens may be due to biotransformation in the skin, which reduces the systemic availability of the parent compound substantially following dermal absorption [20, 21]. Thus, actual absorbed doses of the parent compounds reaching systemic circulation in the biomonitoring dataset are not known. Only the amount of parent compound (free plus conjugated) excreted in urine was measured, which is the minimum amount that was absorbed. For the parabens, which may undergo hydrolysis or Phase I oxidative metabolism following systemic dermal absorption, the amount recovered in urine is likely to underestimate the actual absorbed amount to some degree, although likely to a lesser degree following dermal absorption than following oral absorption [13]. For these compounds, the estimated “maximum” absorbed dose calculated by applying the urinary excretion fraction observed following controlled oral dosing is likely to bracket, but also likely to overestimate, systemically absorbed parent compound. For triclosan, this may be less of an issue, since the majority of administered triclosan is recovered in urine as parent compound (either free or conjugated, both of which are measured in the urinary biomarkers) [14].

A few previous efforts have evaluated exposure models in comparison to population biomonitoring data. Bakker et al. [7] evaluated triclosan exposures from both personal care products and other consumer products and found that even a refined Tier 2 approach substantially over-predicted exposures that were estimated based on population biomonitoring data. This effort used in the RIVM PACEM (Probabilistic Aggregate Consumer Exposure Model) was evaluated for diethyl phthalate and cyclic siloxane (D5) [9, 10], comparing predicted vs. observed urinary biomarker distributions due to uses of personal care products in a population with unknown product uses and unknown time between product use urinary spot sample collection. The modeling employed population distributions for habits and practices as well as an integrated physiologically-based pharmacokinetic model to predict population distributions of biomarker concentrations. This highly refined Tier 2 approach appeared to provide conservative exposure estimates (that is, not likely to underestimate exposures) compared to exposures inferred from biomonitoring data. However, in the current evaluation, Tier 2 modeling did not greatly overestimate the range of observed absorbed doses in the observational dataset. Instead, the Tier 2 models examined here generally captured the range of observed doses, but even the upper end of the predictions from the Tier 2 models did not greatly overestimate the observed absorbed doses, and for triclosan, the observed doses often exceeded the typical range of the model predictions. Because the participants in this study were aware that their product use was being documented and urinary excretion monitored, it is possible that the patterns of product use during the observational period differed from their typical practices. Alternatively, the participants in this study, who were not in any sense a random representation of the general population, may have tended to use products at a higher rate than average, either generally, or specifically during the observation period.

The comparisons presented here of modeled exposures from a variety of Tier 1 and Tier 2 exposure models to the observed absorbed dose data broadly confirm the suitability of such models for providing conservative (Tier 1) or more realistic (Tier 2) estimates of exposure to chemicals included as ingredients of personal care products. The detailed comparisons for the individual participants presented here provide a complementary evaluation to previous evaluations that examined population-wide exposure data (based on biomonitoring) in comparison to modeled exposure distributions (e.g., refs. 7, 9, 10). Such evaluations have the advantage of exposure biomonitoring data over broader populations representing a wide range of behaviors, ingredient prevalence and concentrations, and population characteristics. This dataset and evaluation has the advantage of specific knowledge of the product use ingredients and patterns for the participating individuals, but is based on only a small group of individuals over a very limited time period. Together, the various assessments contribute to the confidence in available models. This analysis also suggests that, rather than relying on a single model, use of multiple models and a range of assumptions may be useful in consideration and characterization of potential exposures to personal care product ingredients. The evaluations of the models and the underlying assumptions used for parameterization (e.g., retention factors) with the observational data provide guidance for future model revisions and assumptions for applications in various contexts.

## References

[1] Csiszar SA, Ernstoff AS, Fantke P, Jolliet O (2017). Stochastic modeling of near-field exposure to parabens in personal care products. J Exp Sci Environ Epidemiol.

[2] International Programme on Chemical Safety (IPCS). Dermal Exposure. Environmental Health Criteria 242. 2014. http://www.inchem.org/documents/ehc/ehc/ehc242.pdf.

[3] World Health Organization (WHO). Principles of characterizing and applying human exposure models. 2005. http://apps.who.int/iris/bitstream/10665/43370/1/9241563117_eng.pdf.

[4] United States Environmental Protection Agency (USEPA). Guidelines for Exposure Assessment. (EPA/600/Z-92/001). Washington, DC. 1992. https://www.epa.gov/risk/guidelines-exposure-assessment.

[5] Dellarco M, Zaleski R, Gaborek BJ, Qian H, Bellin CA, Egeghy P (2017). Using exposure bands for rapid decision making in the RISK21 tiered exposure assessment. Crit Rev Toxicol.

[6] Tiered Aggregate Exposure Assessment (TAGS). 2011. http://www.tags.cperi.certh.gr/.

[7] Bakker M, Biesterbos JWH, Bokkers B, Delmaar C, Dudzina T, N. R, et al. Estimation of realistic consumer exposure to substances from multiple sources and approaches to validation of exposure models. Final report of the Cefic LRI project ETHZ-B7. 2014. http://cefic-lri.org/wp-content/uploads/2014/03/B7_Final-report.pdf [Accessed 1 Aug 2016].

[8] Cowan-Ellsberry CE, Robison SH (2009). Refining aggregate exposure: example using parabens. Reg Toxicol Pharmacol.

[9] Delmaar C, Bokkers B, ter Burg W, Schuur G (2015). Validation of an aggregate exposure model for substances in consumer products: a case study of diethyl phthalate in personal care products. J Exp Sci Environ Epidemiol.

[10] Dudzina T, Delmaar CJ, Biesterbos JW, Bakker MI, Bokkers BG, Scheepers PT (2015). The probabilistic aggregate consumer exposure model (PACEM): validation and comparison to a lower-tier assessment for the cyclic siloxane D5. Environ Int.

[11] Koch HM, Aylward LL, Hays SM, Smolders R, Moos RK, Cocker J (2014). Inter- and intra-individual variation in urinary biomarker concentrations over a 6-day sampling period. Part 2: personal care product ingredients. Toxicol Let.

[12] Smolders R, Koch HM, Moos RK, Cocker J, Jones K, Warren N (2014). Inter- and intra-individual variation in urinary biomarker concentrations over a 6-day sampling period. Part 1: metals. Toxicol Lett.

[13] Moos RK, Angerer J, Dierkes G, Bruning T, Koch HM (2016). Metabolism and elimination of methyl, iso- and n-butyl paraben in human urine after single oral dosage. Arch Toxicol.

[14] Sandborgh-Englund G, Adolfsson-Erici M, Odham G, Ekstrand J (2006). Pharmacokinetics of triclosan following oral ingestion in humans. J. Toxicol. Environ. Health Part A.

[15] European Commission Scientific Committee on Consumer Safety (EC SCCS). The SCCS Notes of Guidance for the Testing of Cosmetic Ingredients and Their Safety Evaluation. 9th Revision. SCCS/1564/15. 2016.

[16] Zhang X, Arnot JA, Wania F (2014). Model for screening-level assessment of near-field human exposure to neutral organic chemicals released indoors. Environ Sci Technol.

[17] Brown TN, Armitage JM, Egeghy P, Kircanski I, Arnot JA (2016). Dermal permeation data and models for the prioritization and screening-level exposure assessment of organic chemicals. Environ Int.

[18] Isaacs KK, Glen WG, Egeghy P, Goldsmith MR, Smith L, Vallero D (2014). SHEDS-HT: an integrated probabilistic exposure model for prioritizing exposures to chemicals with near-field and dietary sources. Environ Sci Technol.

[19] Comiskey D, Api AM, Barratt C, Daly EJ, Ellis G, McNamara C (2015). Novel database for exposure to fragrance ingredients in cosmetics and personal care products. Reg Toxicol Pharmacol.

[20] Cosmetics Ingredient Review (CIR (2008). Final amended report on the safety assessment of methylparaben, ethylparaben, propylparaben, isopropylparaben, butylparaben, isobutylparaben, and benzylparaben as used in cosmetic products. Int J Toxicol.

[21] European Commission Scientific Committee on Consumer Safety (EC SCCS). Opinion on Parabens COLIPA n° P82. SCCS/1348/10. 2011.

[22] European Commission Scientific Committee on Consumer Products (EC SCCP). Opinion on Triclosan COLIPA No. P32 (May 21th, 2009) http://ec.europa.eu/health/ph_risk/committees/04_sccp/docs/sccp_o_166.pdf.

[23] Biesterbos JW, Dudzina T, Delmaar CJ, Bakker MI, Russel FG, von Goetz N (2013). Usage patterns of personal care products: important factors for exposure assessment. Food Chem Toxicol.

[24] Hall B, Steiling W, Safford B, Coroama M, Tozer S, Firmani C (2011). European consumer exposure to cosmetic products, a framework for conducting population exposure assessments. Part 2. Food Chem Toxicol: Int J Publ Br Ind Biol Res Assoc.

[25] Hall B, Tozer S, Safford B, Coroama M, Steiling W, Leneveu-Duchemin MC (2007). European consumer exposure to cosmetic products, a framework for conducting population exposure assessments. Food Chem Toxicol.

